# Prevalence and determinants of antenatal depression among pregnant women in Ethiopia: a systematic review and meta-analysis

**DOI:** 10.1186/s12884-018-2101-x

**Published:** 2018-11-29

**Authors:** Abriham Zegeye, Animut Alebel, Alemu Gebrie, Bekele Tesfaye, Yihalem Abebe Belay, Fentahun Adane, Worku Abie

**Affiliations:** 1 Department of Biomedical Sciences, School of Medicine, Debre Markos University, Debre Markos, Ethiopia; 2 Department of Nursing, College of Health Sciences, Debre Markos University, Debre Markos, Ethiopia; 3 Department of Public Health, College of Health Sciences, Debre Markos University, Debre Markos, Ethiopia

**Keywords:** Prevalence, Antenatal depression, Determinants, Ethiopia

## Abstract

**Background:**

Antenatal depression is more prevalent in low and middle income countries as compared to high income countries. It has now been documented as a global public health problem owing to its severity, chronic nature and recurrence as well as its negative influence on the general health of women and development of children. However, in Ethiopia, there are few studies with highly variable and inconsistent findings. Therefore, the aim of this study was to determine the prevalence of antenatal depression and its determinants among pregnant women in Ethiopia.

**Methods:**

In this systematic review and meta-analysis, we exhaustively searched several databases including PubMed, Google Scholar, Science Direct and Cochrane Library. To estimate the pooled prevalence, studies reporting the prevalence of antenatal depression and its determinants were included. Data were extracted using a standardized data extraction format prepared in Microsoft Excel and transferred to STATA 14 statistical software for analysis. To assess heterogeneity, the Cochrane Q test statistics and *I*^*2*^ test were used. Since the included studies exhibit considerable heterogeneity, a random effect meta- analysis model was used to estimate the pooled prevalence of antenatal depression. Finally, the association between determinant factors and antenatal depression were assessed.

**Results:**

The overall pooled prevalence of antenatal depression, in Ethiopia, was 24.2% (95% CI: 19.8, 28.6). The subgroup analysis of this study indicated that the highest prevalence was reported from Addis Ababa region with a prevalence of 26.9% (21.9–32.1) whereas the lowest prevalence was reported from Amhara region, 17.25 (95% CI: 6.34, 28.17). Presence of previous history of abortion (OR: 3.0, 95% CI: 2.1, 4.4), presence of marital conflict (OR: 7.2; 95% CI: 2.7, 19.0), lack of social support from husband (OR: 3.2: 95% CI: 1.2, 8.9), and previous history of pregnancy complication (OR: 3.2: 95% CI: 1.8, 5.8) were found to be determinants of antenatal depression.

**Conclusion:**

The pooled prevalence of antenatal depression, in Ethiopia, was relatively high. Presence of previous history of abortion, presence of marital conflict, lack of social support from husband, presence of previous history of pregnancy complications were the main determinants of antenatal depression in Ethiopia.

## Background

Depression is one of the earliest and most common diseases of human being. Patients with depression can develop serious disability similar to patients affected by other non-communicable diseases such as diabetes, hypertension and rheumatoid arthritis [1]. Depression is the fourth leading cause of non-fatal diseases burden globally and it accounts about 12% of all total years lived with disability [2]. It has features such as hopelessness, reduced energy, poor thinking, poor concentration, and suicidal attempts during pregnancy, characteristics of antenatal depression [3, 4].

Antenatal depression is a leading mental health problem affecting about one in five women worldwide [5–8]. Compared to high income countries, it is more prevalent in low and middle income countries [7]. The prevalence of antenatal depression is estimated to be 15.6% in low and middle income countries [5]. The disease has now been contemplated to be a global public health problem because of its severity, recurrence and chronic nature as well as its negative effect on the overall health of women and development of children [9–12].

The development of depressive symptoms increases significantly during prenatal period [12, 13] and clinically important depressive symptoms are common in mid and late trimesters [4]. Numerous studies have reported that depressive symptoms are more common during pregnancy than during the postnatal period [12–16].

Some factors are purported to be associated with antenatal depressive disorders such as unplanned pregnancy, financial problems, previous abortion, poor husband support and previous obstetric complications [17–19]. In addition, previous psychiatric problems, chronic medical disorders and poor antenatal service have been reported [8, 20–23]. It is also documented that antenatal depressive disorders result in postnatal depressive disorders, poor rearing capacity in children as well as high recurrent spontaneous abortions [6]. Preterm delivery, impaired postnatal growth, intrauterine growth retardation, frequent infant diarrheal diseases as well as poor social functioning and immune related disease [24] are also the results of unmanaged antenatal depressive disorders [25, 26]. Depressed mood during pregnancy has also been associated with poor attendance at antenatal clinics, substance misuse, low birth weight, and preterm delivery [27, 28].

A systematic review and meta-analysis research done in developed countries presented that the prevalence rates of depression were 7.4% (2.2–12.6%), 12.8% (10.7–14.8%), and 12.0% (7.4–16.7%) for the first, second, and third trimesters, respectively [29]. A systematic review from low- and middle income countries showed that the mean weighted prevalence of antenatal depression was 15.6% (95% CI: 15.4–15.9%) [7]. High prevalence of antenatal depression has been observed from developing countries i.e. 29% in Bangladesh [22], 25% in Pakistan [30], 20.2% in Brazil [31], 39% in South Africa Cape Town [21], 38.5% in South Africa KwaZulu-Natal [32] and 39.5% in Tanzania [18].

In Ethiopia, different studies were conducted to determine the prevalence and factors associated with antenatal depression. Those studies reported that the prevalence of antenatal depression among pregnant women ranges from 11.8 to 31.2% [16, 33–40]. The findings from these small studies were highly variable and inconsistent. Therefore, the main aim of this systematic review and meta-analysis was to determine the pooled prevalence and associated factors of antenatal depression in Ethiopia using available studies. The findings of this study will have an input to policy makers and program planners in the design of appropriate interventions to decrease antenatal depression in the country. In addition, the study will have an importance for clinicians and future researchers in related topics.

## Methods

### Search strategy and study eligibility

All published or unpublished original studies conducted in Ethiopia reporting the prevalence of antenatal depression until 12th of November, 2017 were included in this review. The search was limited to English language and human studies.

Those articles which were not fully accessed after we tried to contact the primary author two times through email were excluded because we were unable to assess the quality of each article without accessing the full text.

The current systematic review and meta-analysis was conducted based on the review of different literatures. The international databases including PubMed, Google scholar, Science Direct, HINARI, EMBASE, Cochrane library were exhaustively searched. In addition, reference lists of already identified articles were also searched to retrieve more relevant studies. Preferred Reporting Items for Systematic Reviews and Meta-Analyses (PRISMA) [41] was used as a guideline for a rigor. The search was carried out using the following keywords by Boolean operator: “Prevalence” OR “Epidemiology” AND “antenatal depression” OR “depression during pregnancy” OR “prenatal” OR “Pregnancy” OR “Pregnant women” AND “Ethiopia”. The search was conducted from the 1st of October to the 12th of November, 2017.

### Operationalization of outcome measurements

The primary outcome of this study was the prevalence of antenatal depression. The prevalence was calculated by dividing the number of women who have depression to the total number of women who have antenatal follow up. The second outcome considered in this systematic review and meta-analysis was a determinant of antenatal depression. For analysis of the second outcome (a determinant), the data extraction format was prepared for each specific determinant (presence of previous history of abortion, presence of marital conflict, lack of social support from husband and previous history of pregnancy complications). We selected these variables because they are the most frequently reported predictors by the studies included in this met-analysis. In this study, we considered a variable as a determinant (presence of previous history of abortion which, in this review, refers to both spontaneous and induced abortions, presence of marital conflict, lack of social support from husband and previous history of pregnancy complications) if at least two or more studies reported them as a determinant. For each determinant, to calculate the odds ratio, the data were extracted from the primary studies in the form of two by two tables. Any disagreement between the two authors during the data extraction was discussed and solved through consensus.

### Data extraction and quality assessment

To extract the necessary data from the studies, the full texts of the articles were assessed by the two authors (AZ and AA).

Any discrepancy was resolved by discussion, and the following information was independently extracted from each study by the two authors (AZ and AA) using a standardized data extraction format: primary author, publication year, region of the study (study site in the country), sample size, screening tool used, response rate and reported prevalence of antenatal depression.

The quality of eligible studies was assessed against predefined inclusion criteria. The quality assessment tool for cross sectional studies known as Newcastle-Ottawa Scale [42] was used. The tool mainly assesses the following parameters: sampling strategy, sample size, inclusion/exclusion criteria, screening tools used to assess the outcome and statistical models used to identify the risk factors. Generally, differences between reviewers were resolved by discussion and consensus. Finally, studies with a scale of ≥6 out of 10 scales were considered as high quality.

### Statistical analysis

The necessary information from each original study was extracted by using a format prepared in Microsoft Excel spreadsheet. Then the data were transferred in to STATA 14 statistical software for analysis. Binomial distribution formula was used to calculate the standard error of each original study. Computed values of chi-square, I^2^ and *p*-values were used to assess heterogeneity among the studies [43]. Due to a considerable heterogeneity among studies (I^2^ = 92.5%, *p* < 0.01), random effects model was used to estimate the Der Simonian and Laird’s pooled effect. Subgroup analysis was done based on the region where the studies were conducted and based on a screening tool used to minimize the random variations between the point estimates of the primary studies. In addition, univariate meta regression was undertaken by taking the sample size and year of publication to identify the possible source(s) of heterogeneity but none of them was statistically significant. Furthermore, publication bias was assessed by using funnel plot inspection as well as Egger’s and Begg’s tests at 5% significant level [44]. Significant publication bias was observed in the tests. As a result, Duval and Tweedie’s Trim and Fill analysis was done to adjust the final pooled effect size. Sensitivity analysis was also performed after defining high quality studies as having an assessment score greater than/equal to 6 and by using the publication status of the studies. Point prevalence as well as their 95% confidence interval was presented in forest plot. In this plot, the size of each box indicates the weight of the study, while each crossed line refers to 95% confidence interval. To identify possible determinants, pooled effect was articulated in the form of odds ratio.

## Results

### Explanation of identified studies

**Fig 1:**
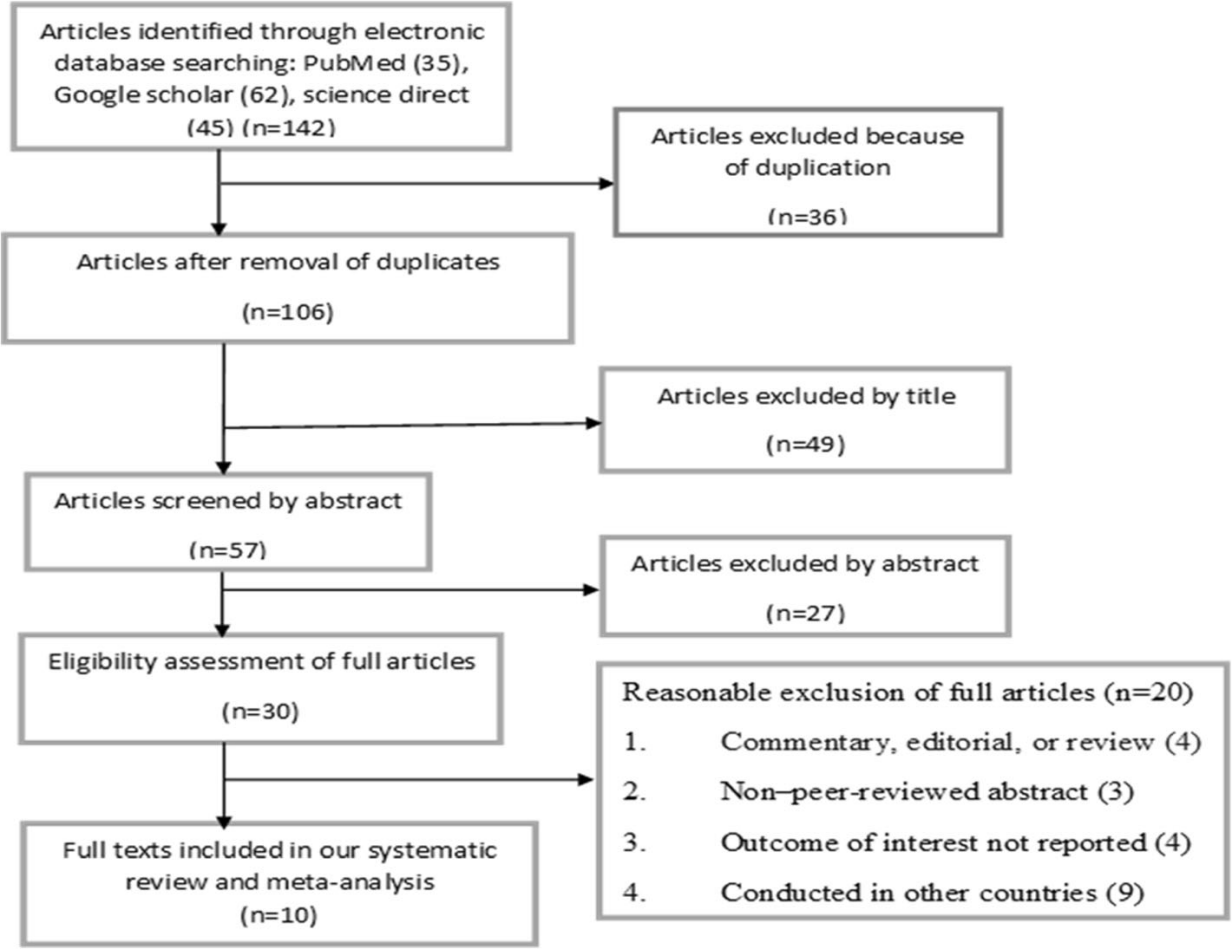
Flow diagram showing the procedure of selecting studies for meta-analysis of prevalence and determinants of antenatal depression in Ethiopia, 2017

Figure 1 shows the searching processes of the original studies for this review. Initially, 142 retrievals were identified from the electronic databases of MEDLINE/PubMed, Google scholar, Science Direct, HINARI, EMBASE, Cochrane Library, and reference lists of previous relevant studies. After excluding 36 articles because of duplication, the remaining articles were screened based on the pre-set eligibility criteria. Then, after reviewing the titles and abstracts of 106 retrievals, duplicated ones were excluded. From these, 49 articles were found irrelevant for this review in terms of outcome of interest. Only 10 articles were considered eligible and included in this meta-analysis after reading the full texts and applying the inclusion criteria,

Among 19 full text articles accessed, we excluded nine articles as they were carried out in other countries: in developed countries [29], in low- and middle income countries [7], in Australia [5] in Bangladesh [22], in Pakistan [30], in Brazil [31], in South Africa Cape Town, in South Africa KwaZulu-Natal [32], in Nigeria [13] and in Tanzania [18].

### Characteristics of original articles

Table 1 summarizes the characteristics of 10 original articles included in this systematic review and meta-analysis. Regarding the study design, nine of the articles were cross sectional whereas one study is prospective cohort. The sample size of included studies varied from 187 (in Addis Ababa, Zewditu Memorial Hospital) to 1311 (in Southern Nations and Nationalities of Ethiopia, Sodo District). These studies were conducted between 2013 and 2017. In this meta-analysis, a total of 4983 Ethiopian women participated to estimate the pooled prevalence of antenatal depression. From nine regions of the country, 10 studies were conducted in six regions; Amhara [35, 36], Tigray [16] and Southern Nations, Nationalities and Peoples (SNNP) [40], Oromia [34, 37, 39], Addis Ababa [33] (Hanlon C: A Screening for Antenatal depression: a formative study for development of a perinatal mental health liaison service in Zewditu hospital, unpublished) and Afar [35]. The highest prevalence (31.2%) of antenatal depression was reported from a study conducted in Adama town (Oromia region) [34] on the other hand, the lowest prevalence (11.8%) was reported from a study done at Debre Tabor Hospital (Amhara region) [36]. Moreover, the response rate of the included studies ranges from 92.8% [35] to 99% [39], and almost all studies have good response rate. Regarding the publication status of the articles, two of 10 studies were unpublished articles which were accessed by communication with study investigators (Hanlon C, A Screening for Antenatal depression: a formative study for development of a perinatal mental health liaison service in Zewditu hospital, unpublished) [45], but eight of the studies were published articles in peer reviewed reputable journals. The quality score of the articles ranged from 5 to 8 out of 10 points (Table 1).

**Table 1:** List of studies included to show the prevalence and determinants of antenatal depression, 2017, Ethiopia

Region	Institution	Author	Publication year	Study design	Screening tool	Sample size	Response rate (%)	Quality score (10 pts)	Prevalence (95% CI)
Addis Ababa	Public health centers	Biratu and Haile [33]	2015	Cross- sectional	EPDS	422	93.13	6	24.94(20.66–29.21)
	Zewditu Memorial Hospital	Asmeret Andebirhan (Hanlon C: A Screening for Antenatal depression: a formative study for development of a perinatal mental health liaison service in Zewditu hospital, unpublished)	2014	Cross- sectional	PHQ-9	187	98.9	7	30.27(23.65–36.89)
Amhara	Debre Tabor hospital	Bisetegn et al. [36]	2016	Cross- sectional	EPDS	543	97	6	11.8(9.05–14.55)
	Gonder university hospital	Ayele A et al. [35]	2016	Cross- sectional	21-Item BDI	418	92.8	6	22.94(18.75–27.12)
Oromia	Gilgel Gibe Dam area	Dibaba et al. [39]	2013	Prospective cohort	EPDS	627	99	5	19.94(16.823.08)
	Shashemene hospital	Gemeta WA et al. [37]	2014	Cross- sectional	EPDS	660	98.3	7	25.6(22.24–28.96)
	Adama town hospital	Sahile et al. [34]	2017	Cross- sectional	21-Item BDI	243	95	7	31.17(25.2–37.14)
SNNPR	Sodo District	Bitew et al. [40]	2017	Cross- sectional	PHQ-9	1311	95.5	8	29.5(26.97–32.03)
Tigray	Maichew Town	Tilahun B. et al. [16]	2017	Cross- sectional	EPDS	209	93.8	5	31.12(24.64–37.60)
Afar	pastorals areas	Abebe y et al. [45]	2017	Cross- sectional	21-Item BDI	363	98.3	7	17.9(13.90–21.88)

*Abbreviations*: *BDI* Beck Depression Inventor, *PHQ-9* 9-item Patient Health Questionnaire, *EPDS* Edinburgh Postnatal Depression Scale

### Meta-analysis

**Fig. 2:**
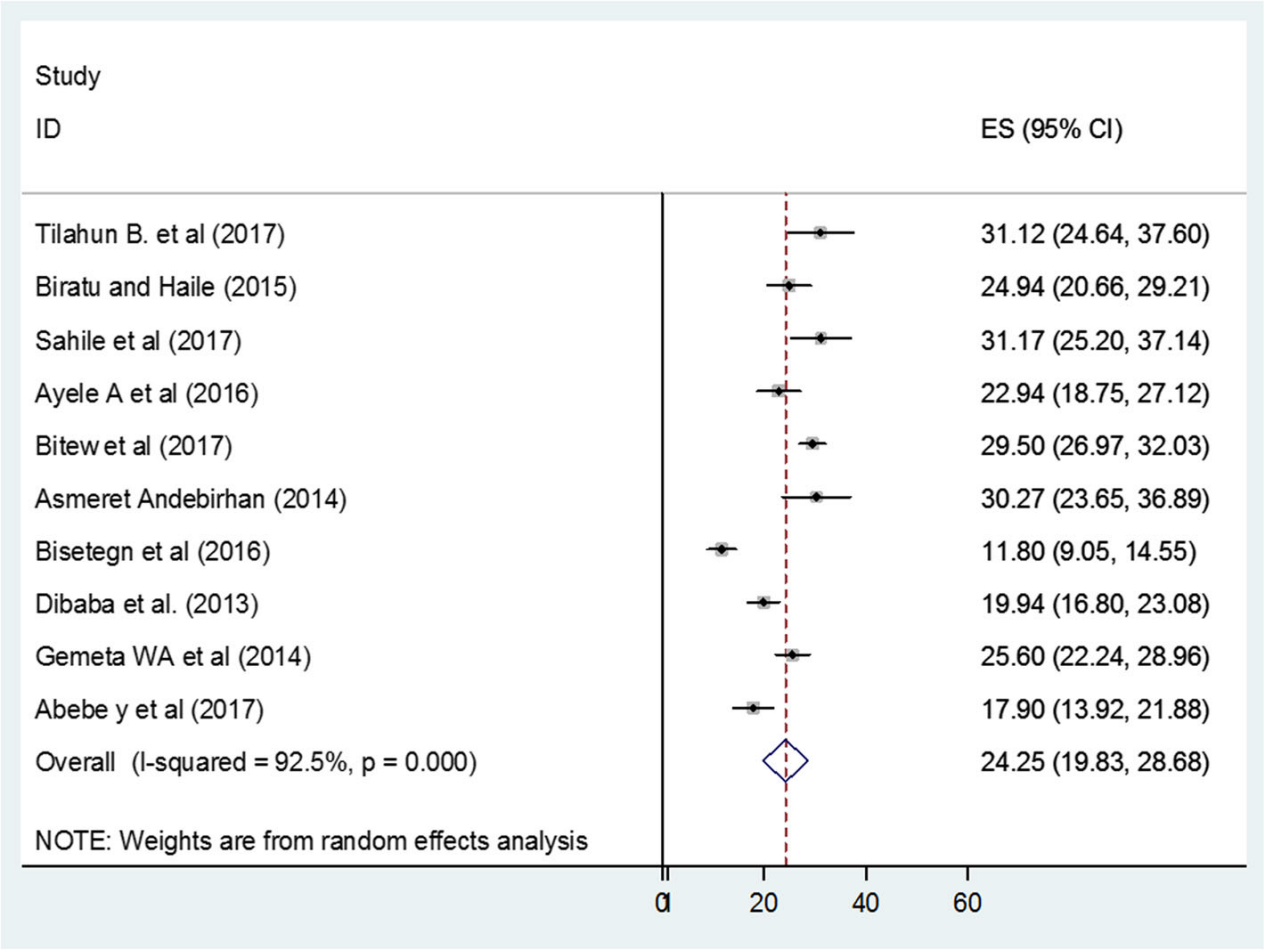
The pooled prevalence of antenatal depression in Ethiopia, 2017

The result of this meta-analysis indicated that the overall pooled prevalence of antenatal depression, in Ethiopia, was 24.2 (95% CI: 19.8, 28.7) (Fig. 2). As presented in Fig. 2, the I^2^ test result showed considerable heterogeneity (I^2^ = 92.5%). Therefore, we employed a random effect meta-analysis model to estimate the pooled prevalence of antenatal depression. To identify the possible sources of heterogeneity, different factors associated with the heterogeneity such as publication year and study design were investigated by using univariate meta-regression models, but none of these variables was found to be statistically significant (Table 2).

**Table 2:** Related factors with heterogeneity of antenatal depression prevalence in Ethiopia in the current meta-analysis (based on univariate meta-regression)

Variables	Coefficient	P-value
Publication year	− 0.536	0.94
Sample size	0.0022	0.92

**Fig. 3:**
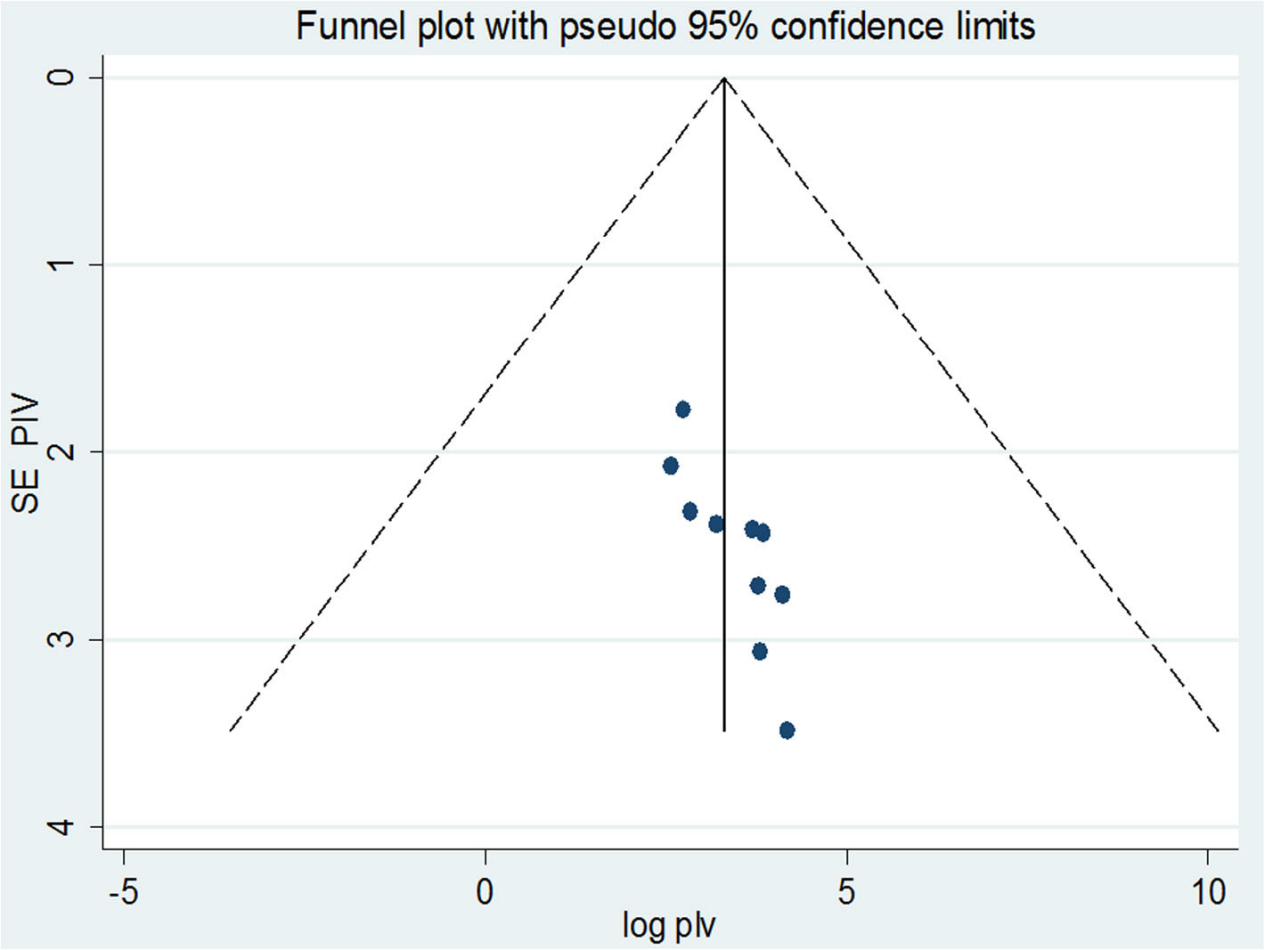
Funnel plots to test the publication bias of the 10 studies

**Fig. 4:**
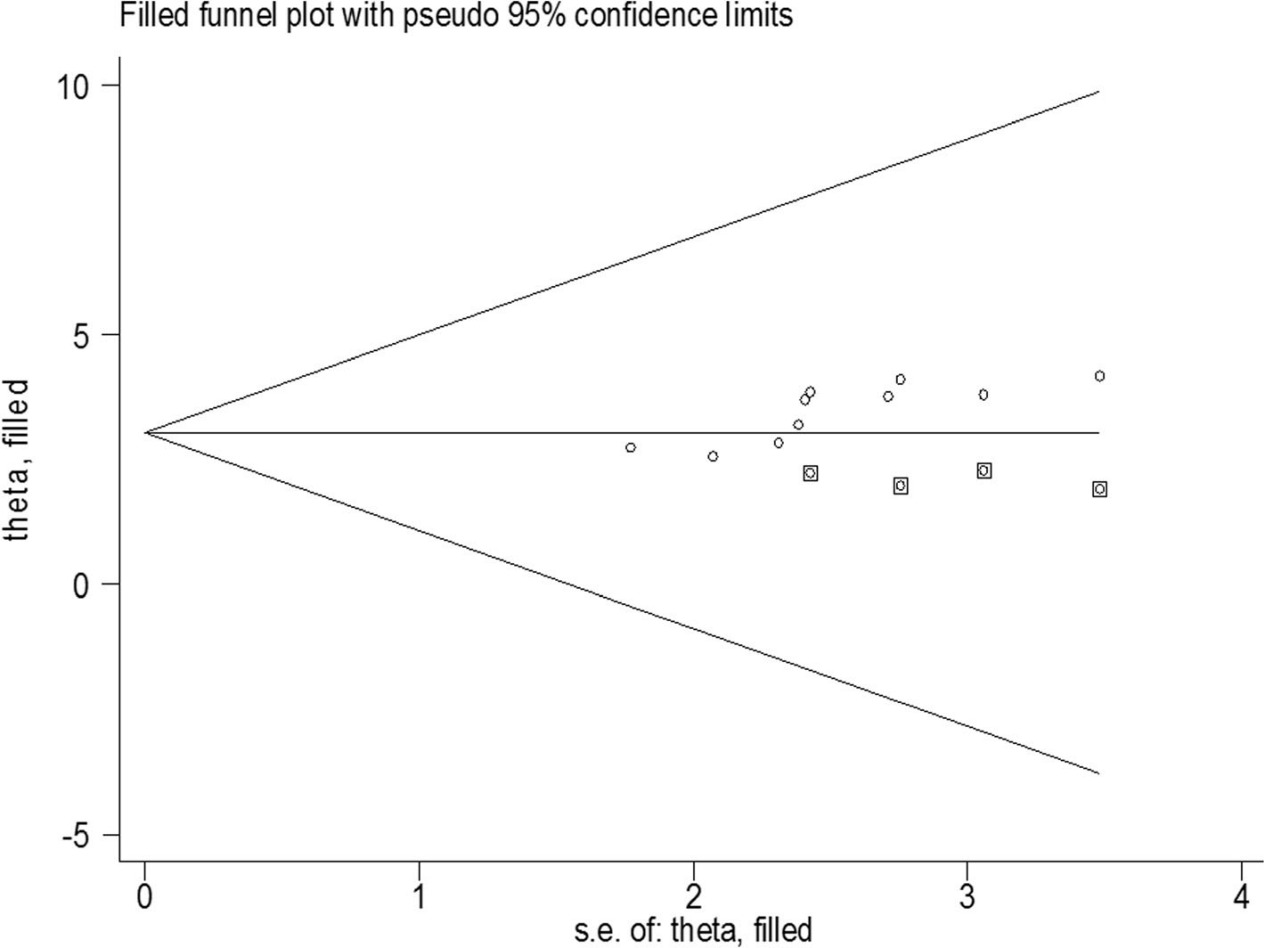
Duval and Tweedie’s Trim and Fill analysis

To identify the extent of publication bias, we assessed the funnel plot for a symmetry by visual inspection for antenatal depression, and it appeared quite asymmetrical (Fig. 3) indicating the presence publication bias. Egger’s and begg’s test also revealed evidence of publication bias (*p* < 0.05, p < 0.05, respectively). Therefore, Duval and Tweedie’s Trim and Fill analysis was done (Fig. 4).

### Sensitivity analysis

By eliminating data from the meta-analytic model in order to examine influence of low quality and high-bias-risk studies on the overall estimate, sensitivity analyses were performed for all of the studied effect sizes. Only eight studies of higher quality (*n* = 8, 80%) were included in the sensitivity analyses as presented in Table 1. Accordingly, the prevalence of antenatal depression among high quality studies was found to be 24.08 (95% CI: 18.78, 29.38). Thus, quality scores did not significantly affect overall prevalence estimate of antenatal depression, and heterogeneity remained significant.

In order to examine influence of unpublished studies, sensitivity analyses were also performed for all of the studied effect sizes by removing data from the meta-analytic model. Hence, two studies were unpublished; eight studies were included in the sensitivity analyses. The sensitivity analyses using random effects model resulted in the prevalence of antenatal depression to be 24.41 (19.31, 29.50). Therefore, publication status did not significantly affect overall prevalence estimate of antenatal depression, and heterogeneity keep on significant.

### Subgroup analysis

In addition, in this meta-analysis, we performed subgroup analysis based on the region where the studies were conducted (Ethiopia has a federal state sub-divided into socio-cultural and ethno-linguistically based regional states and chartered cities) and the screening tool used. Accordingly, the highest prevalence was observed in Addis Ababa region with a prevalence of 26.98% (21.9–32.1) followed by others (26.0%, 17.7, 34.4) and Oromia region (25.1%, 19.4, 30.9) (Table 3).

**Table 3:** Subgroup prevalence of Antenatal depression in Ethiopian, 2017 (*n* = 10)

Variables	Characteristics	Number of studies	Total No_−_ participants	Prevalence with 95% CI
Region	Oromia	3	1530	25.12 (19.36,30.88)
	Amhara	2	961	17.25(6.34,28.17)
	Addis Ababa	2	599	26.98 (21.90–32.06)
	Others	3	1883	26.04 (17.71,34.36)
Screening tool	EDPS	5	2461	22.39% (15.97,28.80)
	21-BDI	3	1024	23.69% (16.81,30.57)
	9-PHQ	2	1498	29.60%(27.24,31.96)

*Abbreviations*: *BDI* Beck Depression Inventor, *PHQ-9* 9-item Patient Health Questionnaire, *EPDS* Edinburgh Postnatal Depression Scale

With regard to the screening tools used to measure the outcome variable, the prevalence of ante natal depression was slightly higher in studies done using Patient Health Questionnaire (PHQ) 29.60% (27.24,31.96) followed by studies done by Beck’s Depression Inventory scale (BDI) 23.69% (16.81,30.57) and Edinburgh Postnatal Depression Scale 22.39% (15.97,28.80) (Table 3).

### Determinants of antenatal depression

**Fig. 5:**
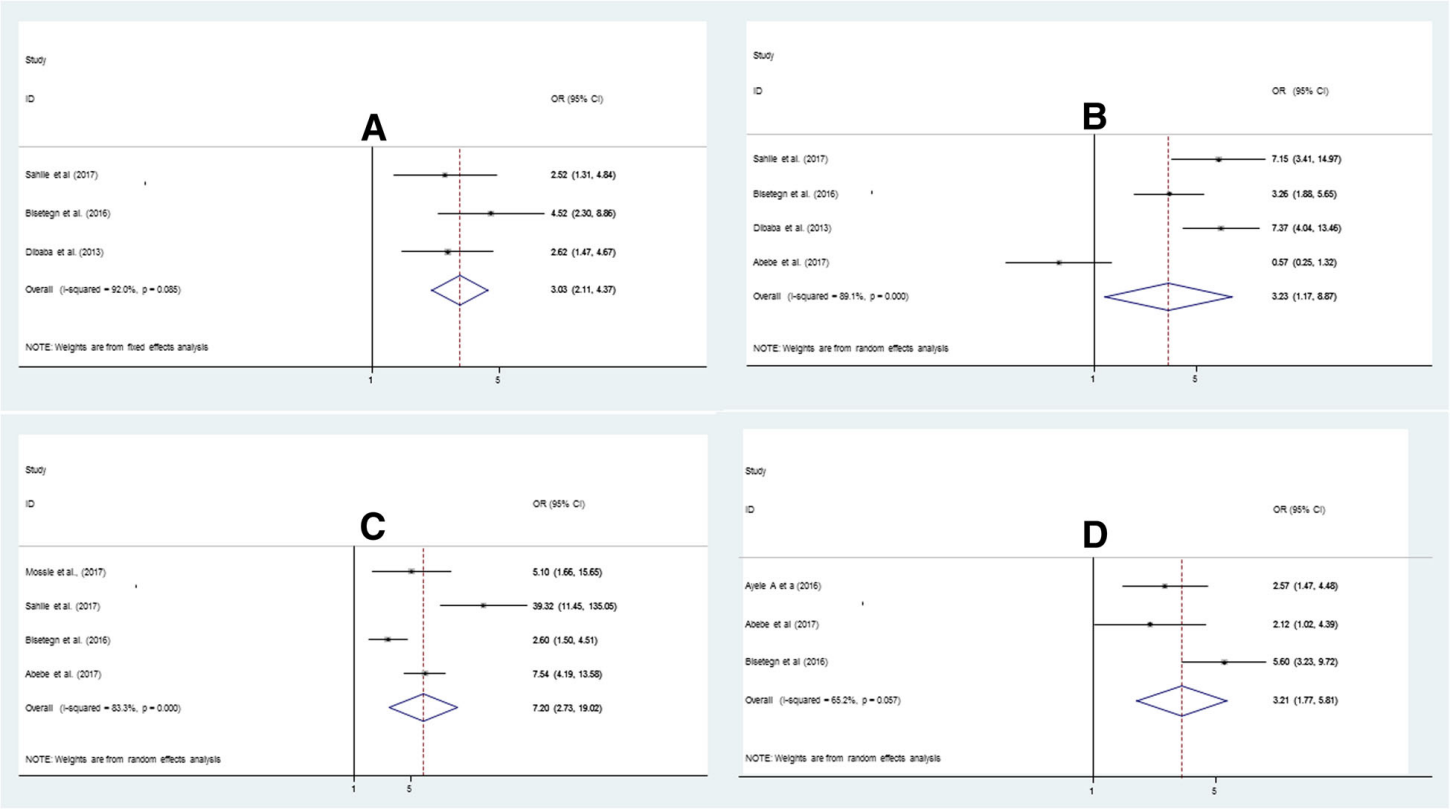
Forest plot depicting pooled odds ratio (log scale) of the associations between antenatal depression and its purported determinants (**a**: previous history of abortion **b:** Presence of marital conflict **c**: lack of social support from husband **d**: previous history of pregnancy complication)

Six studies [16, 34–36, 39, 45] were included for the analysis of risk factors for antenatal depression. Accordingly, four risk factors had data that could be used in the quantitative meta-analysis. The pooled odds ratios ranged from 3.0 (history of abortion) to 7.2 (marital conflict). Greater heterogeneity was observed among studies evaluating previous abortion history, lack of social support from husband, marital conflict and previous history of pregnancy complications (Fig. 5a-d).

### Previous history of abortion and antenatal depression

A total of three articles with 1413 participants were included to assess the association of previous abortion history and antenatal depression [34, 36, 39]. The findings showed a significant association between previous abortion history and antenatal depression. Those who had previous history of abortion were 3 times more likely to develop antenatal depression (OR: 3.0, 95% CI: 2.1, 4.4) (Fig. 5a) as compared to those who had no previous history of abortion.

### Marital conflict and antenatal depression

A total of four articles with 1358 participants were included to determine the association between marital conflict and antenatal depression [16, 34, 36, 45]. Those mothers who had marital conflict were 7 times more likely to develop ante natal depression as compared to those who had no marital conflict (OR: 7.2; 95% CI: 2.7, 19.0) (Fig. 5b).

### Lack of social support from husband and antenatal depression

Four articles with a total of 1776 participants were included in the analysis to detect the association between lack of social support from husband and antenatal depression [34, 36, 39, 45]. Individuals who reported to have lack of support from their husband were 3.23 times more likely to have antenatal depression (OR: 3.2: 95% CI: 1.2, 8.9) (Fig. 5c).

### Previous history of pregnancy complications and antenatal depression

Three articles with a total of 1324 participants were included in this analysis [35, 36, 45]. Those mothers who experience previous pregnancy complications were 3.21 times more likely to develop antenatal depression (OR: 3.2: 95% CI: 1.8, 5.8) in current pregnancy (Fig. 5d).

## Discussion

The present systematic review and meta-analysis was conducted to address two research objectives. The first objective was to estimate the pooled prevalence of antenatal depression among pregnant women in Ethiopia. The second objective was to identify the determinants of antenatal depression. The result of this meta-analysis indicated that the overall prevalence of antenatal depression was relatively high. Since the progress of depression has been interrelated to a greater risk of upcoming depressing experiences and countless long-term morbidity, these findings may affect the long-term health of mothers including their offspring (17, 47).

The overall pooled prevalence of antenatal depression in Ethiopia was 24.25 (95% CI: 19.83, 28.68). This finding was higher than the prevalence estimated in low and middle income countries [5], a systematic review and meta-analysis studies done in the developed countries [29] and low- and middle income countries [7]. But, still there are a lot of studies which verify that this pooled prevalence was lower than studies done in different developing countries like 29% in Bangladesh [22], 39% in South Africa, Cape Town [21], 38.5% in South Africa KwaZulu-Natal [32] and 39.5% in Tanzania [18]. This difference in the findings may reflect differences in participants’ characteristics, sample size, screening tool and publication year. This review included women who had ante natal care follow up in health institution but these studies may include those pregnant mothers who did not have ante natal care follow up. In addition, discrepancy in study methodology and quality of studies may attribute for the difference.

In our systematic review and meta-analysis, random effect models were used bearing in mind the chances of substantial heterogeneity between studies which were confirmed with the Q test. The pooled prevalence of subgroup analysis revealed clear differences on the prevalence of antenatal depression among regions; studies from Addis Ababa region reported high prevalence of antenatal depression. This discrepancy is due to the fact that the methodological differences between studies and diverse measurement tools might attribute to the difference in the prevalence of depression among these regions. Life style activities in Addis Ababa region are associated with socio demographic and economic differences that might also attribute for the difference in prevalence of antenatal depression between these studies and the studies from other regions.

In inferring the results of this systematic review and meta-analysis, it is important to remind that the vast communal of participants were evaluated through self-report inventories that measured depressive symptoms rather than gold-standard screening clinical interviews for most depressive disorder. In addition, even if each self-report measure of depressive symptoms has limitations, there is an indication that the lack of anonymity in formal screening assessments may compromise precise assessment of sensitive personal information such as depressive symptoms. Difference in study sample size and publication year did not contribute importantly to the observed heterogeneity in the data. But, some studies used screening instruments in nonstandard ways (eg, with cutoff scores that have not been validated).

The results of this meta-analysis highlighted common risk factors for antenatal depression. Even though there is significant heterogeneity in ORs, a lot of studies had OR > 3, indicating an increased risk for antenatal depression. The combined results of the six studies about the risk factors for antenatal depression indicated that four factors: previous history of abortion, marital conflict, lack of social support from husband and previous history of pregnancy complications were the main determinants for antenatal depression in Ethiopia. These findings are similar with the findings of a study conducted in low- and middle income countries [7] and study done in Bangladesh [22].

In this study, antenatal depression was 3 times more prevalent in women who had history of abortion than who had not history of abortion. This finding was consistent with a study in Brazil [31] and a systematic review conducted in high income countries [29]. The strong association observed in our study might reflect an increased vulnerability to depression which may be intensified by lifestyle changes (e.g. sleeping and eating patterns) as well as physical changes (e.g. abortion -related symptoms and limitations) during previous pregnancy.

Experiencing marital conflict was found to be a significant factor associated with depression during pregnancy. Those women who had marital conflict were about seven times more likely to have antenatal depression as compared to women who had no marital conflict. This finding was consistent with other studies [17, 35, 45]. This might be due to the connection between quality of a woman’s intimate partner relationship with physiological and psychological changes that occur during pregnancy. Any factor such as marital conflict which threatens family’s feelings of safety and security has the potential to affect their well-being.

Women who had no social support from their husbands were 3 times more likely to have antenatal depression as compared to those women who had social support. Similar finding was reported from other studies [12, 16, 19]. Social support from their husband, family, partner and friends might help women to cope up with stressful life situations by getting emotional, material, informational and evaluative support during pregnancy. Women whose partners welcome the pregnancy and provided support and encouragement had better mental and emotional health.

Moreover, the current study revealed that there is an association between previous pregnancy complications and antenatal depression. This is consistent with different studies [8, 25, 30, 31]. This might be because of the fact that mothers who were not satisfied by previous pregnancy will pose different psychosocial problems and fear of complications during the course of the pregnancy. In addition, poor understanding and low awareness about pervious pregnancy complications lead to development of depression in progressive pregnancy.

### Limitations of the study

The main limitation of this study was that most of the studies included in this review were cross-sectional in design; it neither represents seasonal variation of depression outcomes nor establishes causal relationship. Second, the data were derived from studies that used different screening tools, the prevalence of antenatal depression could not be precisely determined. In addition, only articles published in English language were considered to include in this review.

It should also be noted that some of the studies included in this review had a small sample size which could affect the estimated report. On the other hand, most samples of this study are from health institutions; as a result, these may not be representative of Ethiopian women. Furthermore, while examining the association between antenatal depression and related factors, we did not adjust our analysis for gestational age (trimester).

## Conclusion

In this meta-analysis, the pooled prevalence of antenatal depression, in Ethiopia, was relatively high. The combined results of the six studies about the determinants of antenatal depression indicated that four factors: previous history of abortion, marital conflict, lack of social support from husband and previous history of pregnancy complications were the main determinants for antenatal depression in Ethiopia. Therefore, based on our conclusions, we recommend that hospital’s antenatal care service should incorporate mental health services. Additionally, health education and early screening of pregnant women as well as training of hospital health professionals on antenatal depression are highly recommended.

## Abbreviations

ANC: Antenatal Care
BDI: Beck Depression Inventor
EPD S: Edinburgh Postnatal Depression
PHQ- 9: 9-item Patient Health Questionnaire
PRISMA: Preferred Reporting Items of Systematic Reviews and Meta-Analysis
SNNPE: Southern Nations, Nationalities and peoples of Ethiopia
WHO: World Health Organization
